# Measurement of the absolute number of photons of the hard X-ray beamline at the Linac Coherent Light Source^1^

**DOI:** 10.1107/S1600577519000250

**Published:** 2019-02-11

**Authors:** Sanghoon Song, Roberto Alonso-Mori, Matthieu Chollet, Yiping Feng, James M. Glownia, Henrik T. Lemke, Marcin Sikorski, Diling Zhu, Stefan Moeller, Hae Ja Lee, Mark S. Hunter, Gabriella Carini, Kai Tiedtke, Ulf Jastrow, Andrey Sorokin, Mathias Richter, Shigeki Owada, Kensuke Tono, Norio Saito, Takahiro Tanaka, Masahiro Kato, Makina Yabashi, Aymeric Robert

**Affiliations:** a Linac Coherent Light Source, SLAC National Accelerator Laboratory, 2575 Sand Hill Road, Menlo Park, CA 94025, USA; b Brookhaven National Laboratory, Upton, NY 11973, USA; c Deutsches Elektronen-Synchrotron, Notkestraße 85, 22607 Hamburg, Germany; d Physikalisch-Technische Bundesanstalt, Abbestraße 2-12, 10587 Berlin, Germany; e RIKEN SPring-8 Center, 1-1-1 Kouto, Sayo-cho, Sayo-gun, Hyogo 679-5198, Japan; f Japan Synchrotron Radiation Research Institute, 1-1-1 Kouto, Sayo-cho, Sayo-gun, Hyogo 679-5198, Japan; g National Institute of Advanced Industrial Science and Technology, NMIJ, Tsukuba 305-8568, Japan

**Keywords:** FEL, hard X-rays, absolute intensity

## Abstract

X-ray free-electron lasers provide intense pulses of coherent X-rays with a short pulse duration. These sources are chaotic by nature and therefore, to be used at their full potential, require that every X-ray pulse is characterized in terms of various relevant properties such as intensity, photon energy, position and timing. Diagnostics are for example installed on an X-ray beamline to specifically monitor the intensity of individual X-ray pulses. To date, these can however only provide a single-shot value of the relative number of photons per shot. Here are reported measurements made in January 2015 of the absolute number of photons in the hard X-ray regime at LCLS which is typically 3.5 × 10^11^ photons shot^−1^ between 6 and 9.5 keV at the X-ray Pump–Probe instrument. Moreover, an average transmission of ≈62% of the hard X-ray beamline over this energy range is measured and the third-harmonic content of ≈0.47% below 9 keV is characterized.

## Abstract 2

Measurement of the absolute number of photons per pulse as a function of X-ray photon energy at the hard X-ray beamline of LCLS is presented. It is estimated by two different detectors and is used to characterize the hard X-ray beamline optics transmission as well as the third-harmonic content of the LCLS radiation.

## 1. Introduction   

Free-electron lasers (FELs) such as the Linac Coherent Light Source (LCLS, Menlo Park, USA) are revolutionary light sources that can provide radiation in the X-ray regime with unprecedented and unique properties (White *et al.*, 2015 ▸). FELs are pulsed sources that can generate intense bursts of X-rays presenting a huge number of almost transversely coherent photons with a very short pulse duration, *i.e.* typically below 100 fs. These new sources have already proven to be extremely valuable tools to enable many investigations of problems in diverse fields of science (Bostedt *et al.*, 2016 ▸).

FEL pulses are typically generated through the self-amplified spontaneous emission (SASE) process, which is inherently stochastic (Kondratenko & Saldin, 1980 ▸; Bonifacio *et al.*, 1984 ▸). This implies that every pulse is different and therefore requires each pulse to be characterized in terms of various parameters such as intensity, position, spectrum and timing. This is currently performed with various diagnostics installed along a beamline and consists for example of the following: beam intensity and position monitors (Feng *et al.*, 2011 ▸; Tono *et al.*, 2011 ▸, 2013 ▸), single-shot spectrometers (Zhu *et al.*, 2012 ▸; Inubushi *et al.*, 2012 ▸) and timing diagnostics (Harmand *et al.*, 2013 ▸; Lemke *et al.*, 2013 ▸). The information provided by these diagnostics, especially the ones provided in a non-destructive way, is critical in the analysis of FEL experimental data, in order to successfully proceed with the necessary normalizing, filtering and/or binning processes.

In most cases, FEL experiments only require a relative measurement of the intensity. However, in some instances, the determination of the absolute number of X-ray photons is of critical importance to support the interpretation of experimental data. In particular, this is for establishing and validating damage thresholds of materials in intense FEL beams, and for the investigation of non-linear X-ray physics phenomena (Doumy *et al.*, 2011 ▸; Glover *et al.*, 2012 ▸; Fuchs *et al.*, 2015 ▸). We also show that this can be used as a tool to monitor the performance of X-ray beamline optics.

In this article we report on the measurements, performed in January 2015, of the absolute number of photons of the LCLS hard X-ray beamline. This work follows the methodology successfully developed for the characterization of the same quantity at the Japanese FEL SACLA (Kato *et al.*, 2012 ▸). This is performed by using two detectors: an X-ray gas monitor detector (XGMD) (Tiedtke *et al.*, 2014 ▸) in tandem with a radiometer (Tanaka *et al.*, 2015 ▸). By correlating these measurements with other diagnostics, we derive the transmission of the LCLS hard X-ray beamline and also provide a characterization of its higher harmonic content.

## 2. Experimental setup   

**Figure 1:**
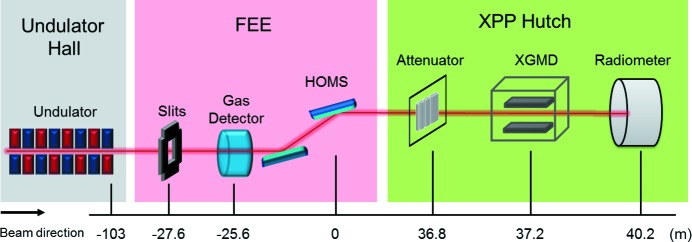
Schematic of the experimental set-up showing the various areas of interest, *i.e.* undulator hall, front-end enclosure (FEE) and XPP instrument hutch, and highlighting the location of relevant optical and detector components, such as the hard X-ray offset mirrors system (HOMS) and X-ray gas monitor detector (XGMD).

The experiment was performed at the X-ray Pump–Probe (XPP) instrument (Chollet *et al.*, 2015 ▸), located on the LCLS hard X-ray beamline (White *et al.*, 2015 ▸). The results of this measurement are, however, useful for all other instruments sharing the hard X-ray beamline: XCS, CXI, MFX and MEC. A schematic of the experimental setup is shown in Fig. 1 ▸. The hard X-ray beam is generated in a long (∼130 m) fixed-gap undulator. It then propagates with a typical divergence of about 2–4 µrad (FWHM) (Turner *et al.*, 2011 ▸) through the front-end enclosure (FEE) area, which contains various beam steering and conditioning optics, and beam diagnostics such as: slits, a gas detector (GD) (Hau-Riege *et al.*, 2008 ▸) and the hard X-ray offset mirrors system (HOMS) (McCarville *et al.*, 2008 ▸; Soufli *et al.*, 2008 ▸). The operating principle of the GD is based on X-ray-induced photo-luminescence of nitrogen gas (Hau-Riege *et al.*, 2008 ▸). The existing GDs are calibrated with the so-called electron beam loss method (Moeller *et al.*, 2011 ▸). The HOMS, located downstream of the GD, is designed for suppressing higher-order harmonics and bremsstrahlung above 25 keV. It consists of two 450 mm × 30 mm (length × height) 50 nm silicon-carbide-coated silicon mirrors reflecting in the horizontal plane with a theoretical 385 mm × 15 mm clear aperture (Soufli *et al.*, 2009 ▸). The typical hard X-ray beam size does not exceed ∼1–2 mm (FWHM) at the mirror location irrespective of the X-ray photon energy. The HOMS therefore fully transmits the X-ray beam in the vertical direction. Both mirrors are located 92.4 m and 103.7 m from the undulator exit, respectively. The grazing incidence angle of both mirrors is by design 1.35 mrad. After the HOMS, the X-ray beam is delivered to all LCLS hard X-ray instruments; in the present case to the XPP instrument which is the instrument located most upstream on the hard X-ray beamline.

The XGMD, provided by the Deutsches Elektronen-Synchrotron (DESY, Hamburg, Germany), was located at the nominal XPP sample position, *i.e.* 37.2 m downstream of the second HOMS mirror. The XGMD provides an absolute measurement of the number of photons per shot by counting ions of rare-gas atoms produced by X-ray photoionization. We used xenon as the target gas at a pressure of ∼10^−2^ Pa. Technical details about the XGMD can be found elsewhere (Tiedtke *et al.*, 2014 ▸). Downstream of the XGMD was installed a radiometer, provided by the National Institute of Advanced Industrial Science and Technology (AIST, Tsukuba, Japan). It provides an average measurement of the absolute number of photons and its technical details have been given by Tanaka *et al.* (2015 ▸).

Various windows and flight path tubes filled with helium or providing a vacuum environment were installed between components to reduce air absorption. Each of these was carefully characterized to properly take into account their contribution to the overall absorption of the X-ray beam. An extensive list of these components and their details are provided in Appendix *A* [App appa]. The measurements were performed at various X-ray energies ranging between 6 and 9.5 keV in SASE lasing conditions with a nominal electron bunch charge of 180 pC, a 40 fs pulse length, and at a 120 Hz repetition rate. In the experimental conditions of this study, the FEE generated X-rays between 1.5 and 3 mJ by the GD at source. Note that in the following the unit mJ will only refer to the numbers provided by the FEE GD.

## 3. Results   

In the following, we present the outcome of the measurements, from which one can obtain detailed information about the X-ray photon energy (*E*_X-ray_) dependence of the average absolute number of photons 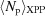 at the sample location of the XPP instrument in a typical experimental configuration. This allows an extrapolation to the average absolute number of photons downstream of the HOMS mirrors 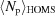, that is relevant for all instruments sharing the same hard X-ray beamline. Details of the hard X-ray beamline transmission are provided together with the characterization of the third-harmonic content of the X-ray beam.

### 3.1. Average absolute number of photons   

XPP is the instrument located the most upstream on the LCLS hard X-ray beamline. It can therefore be used as a benchmark for measuring the average absolute number of photons of the hard X-ray beamline. These measurements can subsequently be used to estimate the same quantity for all other instruments sharing this beamline.

**Figure 2:**
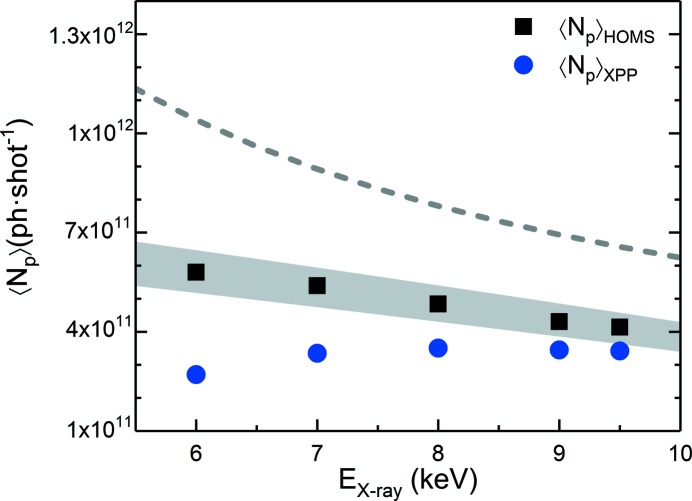
Average absolute number of photons per shot as a function of X-ray photon energy *E*_X-ray_ which is normalized by the FEE GD. The X-ray photon energy dependence of the number of photons, 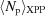, is provided by circles at the XPP sample location under typical experimental conditions. The same quantity circles 〈*N*_p_〉_HOMS_ is extrapolated to the location downstream of the HOMS and is indicated by squares. This is achieved by correcting 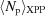 by the transmission of all components located between directly downstream of the HOMS and the XPP sample location. Details are provided in Appendix *A* [App appa]. The dashed line displays the photon energy dependence of the number of photons for a 1 mJ X-ray beam. Error bars of circles are contained within the symbol size. The gray area surrounding the squares indicates a total 10% error margin in transmission correction as a result of the uncertainty of the instrument optics (thickness of one diamond and one kapton window) and calibration error of the FEE GD (Moeller *et al.*, 2011 ▸).

The measurements were performed over the average of many shots, which defines 〈*N*_p_〉 as the average absolute number of photons per shot (note that results are rescaled by 1 mJ of the FEE GD). Fig. 2 ▸ displays with circles the X-ray photon energy dependence *E*_X-ray_ of 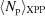, the average absolute number of photons measured by the XGMD at the XPP sample location. It increases with *E*_X-ray_, as expected by the general reduction of X-ray components transmission with *E*_X-ray_, and reaches a plateau at ≈3.5 × 10^11^ photons shot^−1^. A summary of the 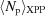 values for each *E*_X-ray_ is provided in the second column of Table 1 ▸.

This number can be further extrapolated at any location along the beamline by properly correcting for the transmission of optical components located upstream of the XGMD. This allows us to extrapolate 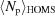 just downstream of the HOMS, as indicated by the squares in Fig. 2 ▸. This number therefore only considers the effect of the HOMS and ranges from 5.8 × 10^11^ to 4.1 × 10^11^ photons shot^−1^ from 6 keV to 9.5 keV. The HOMS transmission is further analyzed and discussed in detail in Section 3.2. A summary of the *E*_X-ray_-dependence of 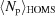 is provided in the third column of Table 1 ▸. Error bars of the circles in Fig. 2 ▸ are contained within the symbol size. The gray area surrounding the squares indicates a total 10% error margin in transmission as a result of the uncertainty of the instrument optics (thickness of one diamond and one kapton window) and calibration error of the FEE GD (Moeller *et al.*, 2011 ▸).

For comparison we also provide the calculated number of photons for a 1 mJ X-ray beam as a function of *E*_X-ray_ as indicated by the dashed line in Fig. 2 ▸. This corresponds to the expected number of photons without the presence of any optical components, and therefore corresponds to a location upstream of the HOMS.

### 3.2. Hard X-ray beamline transmission   

**Figure 3:**
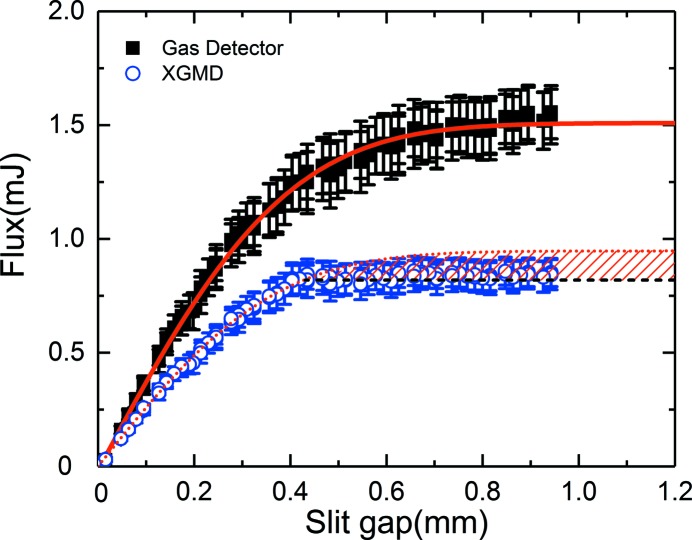
Flux measured simultaneously by the gas detector (GD) (squares) and the XGMD (circles) as a function of horizontal beam size, as defined by the horizontal slit gap. This measurement was performed at *E*_X-ray_ = 6 keV. Solid and dotted lines are simulations of the transmission of a 2D Gaussian beam profile through a one-dimensional slit. The dashed line indicates the average value of the flux measured in the saturation region by the XGMD above ∼420 µm. The dashed area highlights the transmission loss from the HOMS related to its effective active length. The error bars originate from the inherent SASE intensity fluctuation of the beam.

The HOMS delivers the hard X-ray beam up to 25 keV to all LCLS hard X-ray instruments with a theoretical reflectivity of ∼90% for each mirror. However, the X-ray photon energy dependence of the throughput of these mirrors differs from their design values. This originates from various factors such as: the HOMS acts as an effective aperture in the horizontal plane because of the finite size of each mirror but also because of the non-perfect mirror surface, non-perfect reflectivity, and tolerances in grazing incidence angle. The incident beam size also slightly depends on *E*_X-ray_. In order to understand the effective transmission of the HOMS, we collimated the beam horizontally with a slit upstream of the HOMS mirrors and the FEE GD. We then measured the photon flux dependence as a function of the slit gap upstream and downstream of the HOMS with the GD and the XGMD simultaneously, where XGMD was extrapolated from XGMD at XPP. Fig. 3 ▸ presents the flux measured at *E*_X-ray_ = 6 keV by the GD (squares) and the XGMD (circles) as a function of the slit gap. The flux measured by GD (*i.e.* upstream of the HOMS) increases with the slit gap and is reaching a saturation plateau. This plateau corresponds to the case where the slit gap exceeds the beam size in this direction. The solid line is the result of a simulation that was obtained by integrating the transmitted flux of a 2D Gaussian beam profile through a 1D slit, which agrees well with the data.

The XGMD measurement (*i.e.* downstream of the HOMS) increases as a function of the slit gap. The dotted line presents the same simulation as previously, considering that the HOMS mirrors do not clip the beam but including the combined reflectivity of both mirrors. The model describes well the data up to a gap of ∼420 µm from which one can deduce that the effective reflectivity of both mirrors at this energy is ∼65%. For gaps above ≳420 µm a flat plateau is observed as indicated by the dashed line in Fig. 3 ▸. This regime corresponds to the case where the beam size exceeds the effective aperture of the HOMS.

The same measurements were reproduced at different photon X-ray energies (not shown). They all present a similar behavior and lead to a flat plateau of the XGMD data at an average gap 〈*G*〉 ≈ 420 ± 20 µm. This indicates that, with the assumption of an identical incidence angle of α = 1.35 mrad, one can deduce a HOMS effective active length for both mirrors of 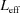 ≈ 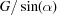 ≃ 311 ± 15 mm. This is much smaller than the physical length (450 mm) and the theoretical clear aperture (385 mm) of these mirrors.

**Figure 4:**
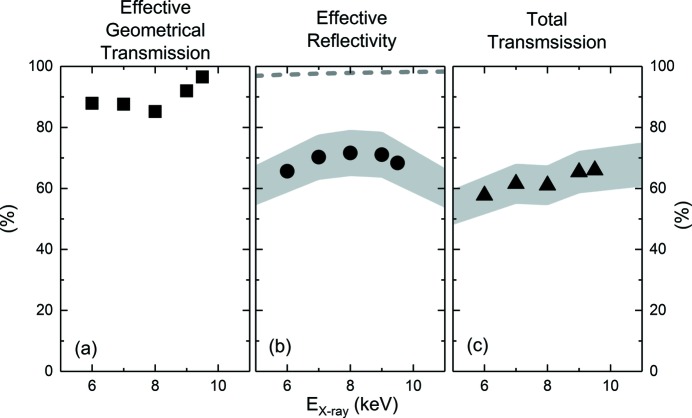
Summary of the outcome of the analysis showing the X-ray photon energy dependence of various parameters of the HOMS: the effective geometrical transmission [(*a*), squares], effective reflectivity [(*b*), circles] and total transmission [(*c*), triangles]. The theoretical effective reflectivity of the HOMS is indicated in (*b*) by the dashed line. Error bars of the squares are contained within the symbol size. The gray area surrounding circles and triangles indicates a total 10% error margin in transmission correction as a result of the uncertainty of the instrument optics (thickness of one diamond and one kapton window) and calibration error of the FEE GD (Moeller *et al.*, 2011 ▸).

From the same measurements, we deduced the total transmission of the HOMS but also estimated their effective geometrical transmission and reflectivity as a function of *E*_X-ray_. The effective geometrical transmission of the mirrors is obtained by calculating the ratio of the flat (dashed line) to saturation (dotted line) plateau of the XGMD data. The results are summarized in Fig. 4 ▸. The total transmission is also indicated in the fourth column of Table 1 ▸.

The effective geometrical transmission displayed in Fig. 4(*a*) ▸ is nearly 90% and is directly related to the HOMS effective active length *L*_eff_, grazing incidence angle α, and incident beam size. The two latter slightly change as a result of the experimental configuration of the FEL and *E*_X-ray_. The HOMS reflectivity is about 65% and the total transmission of the HOMS is more than ∼58% and increases with increasing X-ray energy up to 66%, as displayed in Figs. 4(*b*) and 4(*c*) ▸, respectively. The dashed line in Fig. 4(*b*) ▸ displays the theoretical reflectivity of the HOMS based on their specifications [*i.e.* 1.35 mrad grazing incidence angle and 0.35 nm RMS roughness of silicon mirrors with a 50 nm SiC coating (Barty *et al.*, 2009 ▸)] and strongly exceeds the measurement. This loss of reflectivity could be attributed to damage of the HOMS mirrors surface as a result of their long-term exposure in the FEL X-ray beam.

### 3.3. Characterization of the third harmonic   

Whereas most of the experiments at LCLS have used the first harmonic, because of its large number of photons some third-harmonic content still remains. It is therefore important to evaluate the ratio of the third- to the first-harmonic content. When the first harmonic is tuned up for energies larger than 8 keV, the third-harmonic content is reduced by design by the HOMS mirrors. However, for energies lower than 8 keV there could be a significant amount of third-harmonic signal in the incident beam. This can at times complicate experiments as the third- to first-harmonic ratio will strongly depend on the attenuation used for various steps of the experiments, which results in non-uniform background contributions.

Here we report on the characterization of the *E*_X-ray_-dependence of the third- to first-harmonic intensity ratio *r*_3_(*E*_X-ray_). This is done by measuring the number of photons with both the XGMD and the radiometer as a function of *E*_X-ray_ while varying the attenuation level of the beam. To do so, Si foils of different thicknesses were inserted to tune levels of attenuation of the beam. This results in changes in the ratio of the third to the first harmonic *r*_3_(*E*_X-ray_).

**Figure 5:**
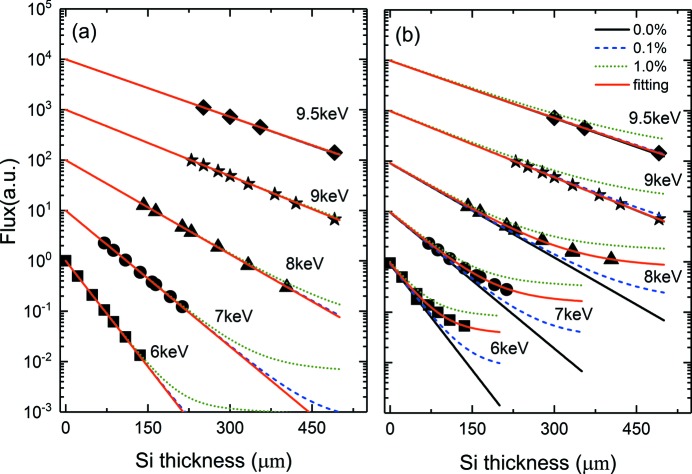
Summary of the X-ray flux measurements for various X-ray photon energies as a function of silicon thickness used for beam attenuation. The measurements obtained by the XGMD and the radiometer are plotted in (*a*) and (*b*), respectively. The various solid, dashed and dotted lines are the result of simulations using equation (1) with a third- to first-harmonic ratio *r*_3_ of 0, 0.1 and 1%, respectively. The solid red line indicates the fit to the data for each *E*_X-ray_, whose results are displayed in Fig. 6 ▸ and are summarized in Table 1 ▸. Each data set has been arbitrarily shifted in the vertical direction for clarity.

Fig. 5 ▸ shows the average pulse energy (symbols) measured as a function of the total Si thickness inserted into the beam for *E*_X-ray_ ranging from 6 to 9.5 keV. Fig. 5(*a*) ▸ summarizes the results obtained with the XGMD, which is most sensitive to the contribution of the first harmonic and is not sensitive to the third-harmonic content in the studied energy range and for the attenuation levels considered here. This low sensitivity to the third harmonic originated from the very low scattering cross section of xenon gas. For example, at 6 keV the ratio of the photoionization cross section of xenon between the third and first harmonic is 5.2 × 10^−2^. Fig. 5(*b* ▸) summarizes the results obtained with the radiometer, which measures the total energy deposited in a target. The absorptance of the radiometer from 0.3 to 40 keV was evaluated using the Monte Carlos simulation and is nearly unity for that energy range (Tanaka *et al.*, 2015 ▸). Therefore it is inherently sensitive to both the first and third harmonic. The measured intensity can be modeled as
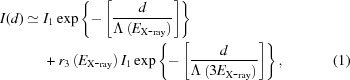
where *I*_1_ is the first-harmonic flux, *r*_3_(*E*_X-ray_) is the third- to first-harmonic ratio at a given first harmonic X-ray energy *E*_X-ray_, *d* is the Si foil thickness and Λ(*E*) is the absorption coefficient of Si for X-rays of energy *E*. In the present case we neglect the possible contribution from the second harmonic (Ratner *et al.*, 2011 ▸).

**Figure 6:**
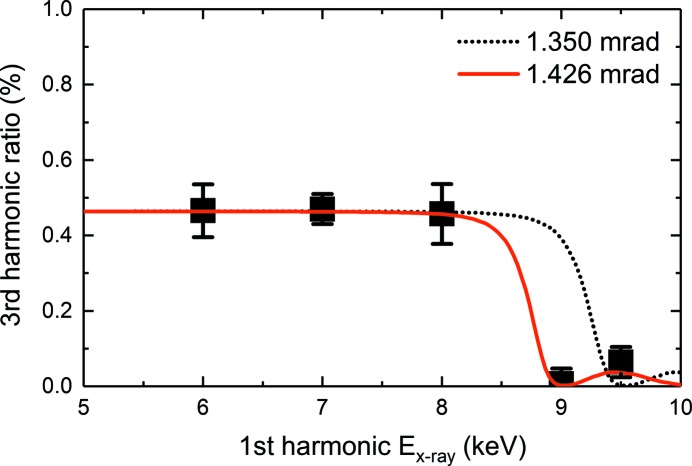
First harmonic X-ray photon energy dependence of the third- to first-harmonic ratio *r*_3_. The dashed line indicates the expected behavior of the HOMS if both angles had an incidence angle of 1.35 mrad. The solid line is a fit to the data and provides an incidence angle of 1.426 ± 0.01 mrad.

The solid, dashed and dotted lines in Fig. 5 ▸ represent the model using equation (1) with the third- to first-harmonic ratios *r*_3_(*E*_X-ray_) of 0, 0.1 and 1%, respectively. As expected for the XGMD measurements one does not observe in Fig. 5(*a*) ▸ any influence of the third- to first-harmonic ratio *r*_3_ on the modeling of the data (for the attenuation levels and *E*_X-ray_ in the present work). In contrast, the radiometer measurements in Fig. 5(*b*) ▸ present a strong deviation from the data in comparison with the model with a third-harmonic ratio *r*_3_ = 0 for energies below 9 keV. The behavior above 9 keV is expected as the HOMS was designed to cut off energies above 25 keV as described previously. The models with *r*_3_ = 0.1% (1%) also seem to underestimate (overestimate) the measured data. Therefore a fit to the radiometer data was performed using *r*_3_ as a fitting parameters and is indicated by the solid line. The results of the fit are plotted as a function of *E*_X-ray_ in Fig. 6 ▸ and are summarized in the fifth column of Table 1 ▸.

The third harmonic content ratio *r*_3_ is observed to be of the order of ∼0.5% for energies up to 8 keV and drops below 0.01% for energies ≈9 keV, which is consistent with the energy cut-off (*i.e.* 25 keV) provided by the HOMS mirrors. The calculated third-harmonic transmission of the HOMS (Henke *et al.*, 1993 ▸) is shown as the dotted line and uses the design incidence angle of 1.35 mrad (Barty *et al.*, 2009 ▸). The solid line is a fit to the data and provides an incidence angle of 1.426 ± 0.01 mrad. It specifically reproduces the slight increase of *r*_3_ observed at 9.5 keV. This bump originates from the thin 50 nm single layer of SiC deposited on the silicon mirror substrates of the HOMS.

With this incidence angle and the earlier result that for slit gaps larger than ≈420 ± 20 µm one observes a transition to a flat plateau of the transmitted flux through the HOMS, we obtain an effective active length for both mirrors of ≈294.5 ± 2.1 mm. This value is similar to the estimate of the effective active length for both mirrors of ≈311 ± 15 mm, previously quoted in Section 3.2. An incidence angle of 1.426 mrad is also in agreement with another study which deduced an incidence angle of 1.45 mrad (Ratner *et al.*, 2011 ▸).

## 4. Summary   

We obtained the absolute number of photons and its X-ray photon energy dependence at the sample position of the XPP instrument. For a typical XPP instrumental configuration it reaches on average 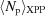 ≈ 3.5 × 10^11^ photons shot^−1^ for the high-energy range of the study and is expressed in units of photons per shot with 1 mJ as provided by the FEE GD. By taking into account the transmission of all components on the beamline, we can extrapolate the average number of photons just downstream of the HOMS mirrors 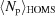, whose location is relevant for all LCLS instruments sharing the same hard X-ray beamline. It ranges from 5.8 × 10^11^ to 4.1 × 10^11^ photons shot^−1^ from 6 keV to 9.5 keV.

We also obtained confirmation that the HOMS mirrors do not operate at their design grazing angle of 1.35 mrad but rather at 1.426 mrad. We further confirm that the mirror reflectivity and their effective active length is well below their design values, and speculate that this could be originating from the long-term exposure effects of both mirrors in the FEL beam.

We were able to characterize the third- to first-harmonic content ratio *r*_3_ of the X-ray beam and its dependence as a function of X-ray photon energy. It is on average 0.47% in this energy range and is ≈50% less than the theoretical predictions of about 1%. In conjunction with the effect of the HOMS mirrors, we have clear evidence of strong third-harmonic suppression for X-ray photon energies larger than 9 keV, but also confirm experimentally a slight increase of *r*_3_ around 9.5 keV. This behavior is consistent with the details of the HOMS mirrors and is clearly attributed to the 50 nm thin SiC coating of each Si mirror.

In an attempt to increase the transmission of the HOMS, LCLS carried out various upgrades to its front-end optics in 2017. The characterization of the new HOMS is ongoing. This study was conducted in January 2015. However, the information presented here provides critical information that can guide the interpretation of LCLS data measured during the User Science Program between October 2010 and December 2016.

## Figures and Tables

**Table 1:** X-ray photon energy dependence of: the average number of photons per shot at the XPP sample location 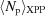 and directly downstream of the HOMS 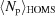 which are normalized by the FEE GD, the HOMS total transmission and the third- to first-harmonic content ratio

	〈*N*_p_〉			
Photon energy	(10^11^ photons shot^−1^)		Transmission	Third-harmonic
(keV)	XPP	HOMS	(%)	ratio (%)
6	2.71 ± 0.12	5.80 ± 0.58	57.7 ± 5.8	0.47 ± 0.07
7	3.35 ± 0.14	5.39 ± 0.54	61.5 ± 6.2	0.47 ± 0.04
8	3.51 ± 0.15	4.84 ± 0.48	61.0 ± 6.1	0.46 ± 0.08
9	3.45 ± 0.18	4.31 ± 0.43	65.3 ± 6.5	0.01 ± 0.04
9.5	3.42 ± 0.14	4.14 ± 0.41	66.0 ± 6.6	0.06 ± 0.04

**Table 2:** Components from the front-end enclosure to the detectors in the XPP instrument that can contribute to the transmission of the X-ray beam

Distance from HOMS (m)	Name	Details	Nominal thickness (µm)	Measured thickness (µm)	Transmission at 6 keV (%)	Transmission at 9.5 keV (%)
−27.6	FEE slit	–	–	–	–	–
−25.6	FEE gas detector	–	–	–	–	–
−11.3	HOMS M1	–	–	–	–	–
0	HOMS M2	–	–	–	–	–
33.9	IPM target	Si_3_N_4_	1	–	99.3	99.8
36.8	Attenuator	Si	–	–	–	–
36.2	Exit window of nominal beamline	Diamond	100 ± 10%	–	69.2	91.3
	Laser in-coupling chamber	Vacuum	–	–	–	–
	Exit window of laser in-coupling	Kapton	25.4 ± 5%	–	95.0	98.7
	Beam path	Air	–	1.25 × 10^5^	71.5	91.8
	Entrance window of XGMD	Diamond	100	109 ± 3	66.9	90.5
37.2	XGMD	–	–	–	–	–
	Exit window of XGMD	Diamond	100	111 ± 3	66.4	90.4
	Beam path	Air	–	1.71 × 10^5^	95.5	98.8
	Beam path window	Kapton	128 ± 5%	–	77.4	93.8
	Beam path	Helium or vacuum	–	1.52 × 10^6^	99.3	99.6
	Beam path window	Kapton	52 ± 5%	–	90.1	97.4
	Beam path	Air	–	9.94 × 10^5^	76.6	99.1
	Entrance window of radiometer	Diamond	100	95 ± 3	70.4	91.7
40.2	Radiometer	–	–	–	–	–

## Appendix A Optical components and description

Table 2 ▸ provides an extensive list of all optical components, air or beam path with their location with respect to the second HOMS mirror. It also includes all necessary information to calculate the transmission of each of these. These transmissions are provided for references for 6 and 9 keV, respectively. For the X-ray photon energies of 8 and 9.5 keV some of the beam paths were substituted with helium at atmospheric pressure instead of being in a vacuum.
